# Clinical and atopic features of patients with primary eosinophilic colitis: an Italian multicentre study

**DOI:** 10.1007/s11739-024-03568-w

**Published:** 2024-03-10

**Authors:** Carlo Maria Rossi, Marco Vincenzo Lenti, Stefania Merli, Antonio Lo Bello, Aurelio Mauro, Andrea Anderloni, Davide Giuseppe Ribaldone, Elisa Marabotto, Marta Vernero, Shirin Djahandideh Sheijani, Daria Maniero, Alessandro Vanoli, Catherine Klersy, Edoardo Vincenzo Savarino, Antonio Di Sabatino

**Affiliations:** 1 Department of Internal Medicine and Medical Therapeutics, University of Pavia, Pavia, Italy; 2 First Department of Internal Medicine, Fondazione IRCCS Policlinico San Matteo, Pavia, Italy; 3 Gastrointestinal Endoscopy Unit, Fondazione IRCCS Policlinico San Matteo, Pavia, Italy; 4 Department of Medical Sciences, University of Turin, Turin, Italy; 5 Gastroenterology Unit, Department of Internal Medicine, University of Genoa, Genoa, Italy; 6 IRCCS Ospedale Policlinico San Martino, Genoa, Italy; 7 Gastroenterology Unit, Azienda Ospedale Università di Padova (AOUP), Padua, Italy; 8 Department of Surgery, Oncology and Gastroenterology (DiSCOG), University of Padua, Padua, Italy; 9 Anatomic Pathology Unit, IRCCS San Matteo, Pavia, Italy; 10 Biostatistics and Clinical Trial Centre, Fondazione IRCCS San Matteo, Pavia, Italy; 11 Clinica Medica I, Fondazione IRCCS Policlinico San Matteo, Università Di Pavia, Viale Golgi 19, 27100 Pavia, Italy

**Keywords:** Atopy, Colon, Eosinophil, Oesophagitis

## Abstract

Eosinophilic colitis (EC) is the rarest among primary eosinophilic gastrointestinal disorders (EGID). EC is underdiagnosed due to its blurred and proteiform clinical manifestations. To explore the clinical and atopic characteristic of EC adult patients, the diagnostic delay, and relapse-associated factors, by comparison with patients with eosinophilic esophagitis (EoE) and irritable bowel syndrome (IBS). EC patients followed-up at four clinics were included, and clinical, histopathological, and laboratory data were retrieved. As control groups, age-matched patients with EoE and IBS were recruited. Allergy tests included skin prick test and serum specific IgE. Diagnostic delay was assessed. Overall, data from 73 patients were retrieved, including 40 with EC (median age 39 years IQR 22.5–59, F:M 2.1:1), 12 with EoE (F:M ratio: 1:5), and 21 with IBS (F:M ratio: 1:0.9). The most common features in EC patients were female sex (67.5%), atopy (77.5%), abdominal pain/distention (70%), diarrhoea (77.5%), and faecal calprotectin elevation (22.5%). Blood eosinophils were elevated in EoE, but not in EC (p < 0.001), while ECP did not differ across the three groups (p = 0.4). The frequency of allergen sensitization reached 25% of patients. Several frequent pan-allergens for this region were present. The overall diagnostic delay was 10 months (IQR 4–15). Factors contributing to a greater diagnostic delay were atopy, weight loss, and a previous misdiagnosis. EC is mostly a diagnosis of exclusion, burdened by a substantial diagnostic delay. In female patients the presence of allergen sensitization, abdominal symptoms and faecal calprotectin elevation should raise the suspicion of EC.

**Supplementary Information:**

The online version contains supplementary material available at 10.1007/s11739-024-03568-w.

## Introduction

Primary eosinophilic colitis (EC) is the rarest and least characterised among the primary eosinophilic disorders of the gastrointestinal tract (EGID) [1, 2]. Its epidemiology has been poorly described, and its pathogenesis remains elusive [2–4]. Allergic triggers have been called into question [3, 4], as for other EGIDs [4, 5], although few studies have addressed this issue. According to the largest, monocentric, retrospective series in EC, the prevalence of atopy was overall low, with a rate of asthma and of food allergy of 6.6% and of 2.8% respectively [6].

A timely diagnosis of EC, which must be made on histopathological grounds, is made difficult due to the unawareness of this entity, the unspecific manifestations, its protean clinical picture related to the various degrees of bowel wall involvement (i.e., mucosal, muscularis or serosal layer), and the patchy distribution of the inflammatory infiltrate. Hence, it is not surprising that EC is thought to be largely underdiagnosed [7, 8]. Further, EC diagnostic and histologic criteria are still disputed [9, 10], and cut-off levels for eosinophil density in gut tracts other than the oesophagus are still debated [11]. Indeed, the diagnosis of EC could be an incidental finding following a colonoscopy performed for the suspicion of other conditions, such as inflammatory bowel disease (IBD) or microscopic colitis. The natural history of the disease remains obscure, ranging from one self-limiting episode of diarrhoea/abdominal pain to a relapsing–remitting course [12]. Factors associated to a relapsing course are unknown.

Starting from these premises, the primary aim of the study was to evaluate the clinical, allergic, and histopathological characteristics of patients with EC by reporting data of a large cohort of adult patients referred to four Italian, academic, tertiary referral centres. The secondary aims were to evaluate EC diagnostic delay, as well as possible patient-dependent and physician-dependent factors associated with a greater delay, and the variables associated with EC relapse.

## Materials and methods

### Patient population and study design

This was a multicentre, observational, retrospective study conducted in four Italian, academic, tertiary referral centres specialized both in the diagnosis and management of patients with primary EGID (Fondazione IRCCS San Matteo, Pavia; Azienda Ospedaliera Università di Padova, Padua; Città della Salute e della Scienza, Torino, IRCCS Ospedale Policlinico San Martino, Genoa) and in the diagnosis and management of allergic disorders.

In November 2022, a face-to-face meeting was held among the study coordinators, including gastroenterology, internal medicine, and allergy consultants (CMR, MVL, ADS, ES), to design the study and to discuss demographic, clinical, laboratory, endoscopic, and histological variables to be included. Afterwards, two other Italian referral centres were contacted and accepted the invitation to take part to the study. The study design was shared and approved by all participants.

We herein retrospectively enrolled all consecutive adult patients who were referred to the participating centres over the last three years (2020–2023) and in whom a diagnosis of EC was confirmed by the treating physician according to the latest, internationally recognized criteria [13]. More in depth, EC diagnosis was made when clinical symptoms were present together with histological evidence of eosinophilic infiltration affecting at least two colon segments. Eosinophil histologic cut-off levels were as follows: cecum and right colon (> 100/HPF), transverse colon and left colon (> 84/HPF), sigma and rectum (> 64/HPF) [11, 14]. We used these stringent criteria as we wanted to make sure that only true primary EC were included. In all cases, three parasite stool cultures were assessed, also including the specific serum antibodies and the fresh stool examination for *Strongyloides stercoralis*; these were negative in all cases. Patients with uncertain histological findings and/or with relevant missing data (i.e., histopathological report not available, or poor biopsy sampling) were excluded. Other exclusion criteria were drug-related colitis, active parasitic infections, immune-mediated gastrointestinal disorders other than EGID (e.g., IBD, microscopic colitis), hyper-eosinophilic syndrome, and eosinophilic granulomatosis with polyangiitis [2, 4]. More precisely, drug-related colitis was ruled out after having excluded the known association with specific drugs as reported in the literature [2, 4]. In order to exclude an upper gastrointestinal involvement, all EC patients underwent un upper gastrointestinal endoscopy; no cases of concomitant eosinophilic esophagitis (EoE), gastritis and/or duodenitis were noticed.

As control groups, adult patients with EoE or irritable bowel syndrome (IBS) evaluated at the allergy clinic were also included from the centre of Pavia. These disorders were chosen as controls, since the first belongs to the spectrum of EGID, but does not involve the colon, while the second involves the colon, but it is not characterized by eosinophilic inflammation and its symptoms may mimic those of EC. Moreover, atopic comorbidities are also present in IBS [15, 16]. The control groups were randomly and consecutively enrolled, and were age-matched with the EC cohort, but not sex-matched, in order to reflect the already known, different, sex distribution across the three diseases. The diagnosis of EoE and IBS were made according to current clinical and/or pathological guidelines [17, 18]. More precisely, the diagnosis of EoE was based upon the presence of consistent clinical symptoms, such as dysphagia, together with histologic infiltration (> 15 eosinophils/HPF) in at least two biopsies from at least two oesophageal segments, in the absence of secondary causes [17]. The diagnosis of IBS was made according to the Rome IV criteria [18]. Indeed, in all patients presenting with diarrhoea biopsies were taken and none had pathological hypereosinophilia.

The diagnostic delay was estimated in all EC patients, and split into patient-dependent -i.e., the time lapse between the onset of symptoms/alterations and seeking medical care- and physician-dependent -i.e., the time lapse between the first medical assessment and the definitive diagnosis- diagnostic delay. Indeed, the overall diagnostic delay was the sum of patient- and physician-dependent delays calculated in months.

Demographic (sex, age, socioeconomic status) and clinical data of patients were extracted and pseudo-anonymised from the electronic hospital records into a pre-defined spreadsheet. Clinical data included clinical manifestations at onset, atopic comorbidities, relevant endoscopic, histopathological, and general laboratory data. Skin prick tests and specific serum IgE to whole extracts and molecular allergens were also included, when available. All data that were not present in the electronic records or in the physicians’ assessment forms were retrieved through a phone call with the patient. Informed consent was obtained from all patients. The study was performed as a clinical audit using routine collected clinical and laboratory data. All participants provided written informed consent which was obtained at the time of the outpatient visit according to the local ethics committee for the use of their data in an aggregated and anonymous format. The study was approved by the ethics committee of the centre of Padua (IMID Register 5370/A0/22). All results are reported according to the STrengthening the Reporting of OBservational studies in Epidemiology (STROBE) recommendations for quality assurance. Due to privacy compliance, the raw data cannot be made public, but can be shared by the corresponding author upon reasonable request.

### Statistical analysis

A sample size was not calculated a priori, given the exploratory, retrospective and descriptive nature of the study and all available patients from the participating centers are included. We report in a supplementary table the minimal detectable difference between the EC cohort and EoE or IBS in a series of potential scenarios, when the type I error if 5% and the power 80%.Continuous data were described with the median and interquartile range (IQR; i.e., 25th-75th percentiles), and categorical data as counts and percent. Comparisons between the three cohorts were performed using the Kruskal–Wallis test for continuous variables and the Fisher exact test for categorical variables. Missing data were excluded from percentage calculation, when specified.

The software Stata 17 (StataCorp, College Station, TX, USA) was used for all computations. A 2-sided p-value < 0.05 was considered statistically significant. For post-hoc comparisons of IBS and colitis vs EoE, significance was set at 0.025 (Bonferroni correction).

## Results

### Demographic data

Overall, data from 73 patients were retrieved from all the participating centres. More precisely, 40 patients with EC (median age 39 years, IQR 22.5–59, F:M ratio: 2.1:1), 12 patients with EoE (median age 36.5 years, IQR 22–51.5, F:M ratio: 1:5), and 21 patients with IBS (median age 35 years, IQR 30–39, F:M ratio: 1:0.9) were included (Table 1). None of the patients with EC displayed any other non-EC EGID. As per study design, age did not differ across the three groups (p = 0.7). Regarding sex, females were instead significantly more frequent among patients with EC, as compared to EoE (p = 0.007). Current or previous smoking was more frequent in patients with EC than in those with IBS (p = 0.014).

**Table 1:** General and demographic characteristics of 40 eosinophilic colitis (EC) patients versus 12 eosinophilic esophagitis (EoE) patients and 21 irritable bowel syndrome (IBS) patients

Variable	Overall cohort n = 73)	EC (n = 40)	EoE (n = 12)	IBS (n = 21)	p-value
Age, years, median (IQR)	36 (26–55)	39 (22.5–59)	36.5 (22–51.5)	35 (30–39)	0.704
Age at diagnosis, years, median (IQR)	35 (25–53)	38 (25–59)	34 (21–49.5)	32 (27–38)	0.600
^£^Gender, n (%)					**0.006**
Female	40 (54.8)	27 (67.5)	2 (16.7)	11 (52.4)	
Male	33 (45.2)	13 (32.5)	10 (83.3)	10 (47.6)	
^**&**^Smoker, n (%)					**0.039**
No	49 (68.0)	23 (57.5)	7 (63.6)	19 (90.5)	
Previous	12 (16.7)	9 (22.5)	3 (27.3)	0 (0.0)	
Current smoker	11 (15.3)	8 (20.0)	1 (9.0)	2 (9.5)	
Family history, n (%)					0.109
Positive family history	8 (15.4)	7 (17.5)	1 (8.3)	-	
Negative family history	44 (84.6)	33 (82.5)	11 (91.7)	-	
Coffee consumption, n (%)					0.317
Yes	41 (57.75)	20 (52.6)	6 (50.0)	15 (71.4)	
No	30 (42.25)	18 (47.4)	6 (50.0)	6 (28.6)	
Alcohol consumption, n (%)					0.350
Yes	17 (23.3)	11 (27.5)	1 (8.3)	5 (23.8)	
No	56 (76.7)	29 (72.5)	11 (91.7)	16 (76.2)	
Years of education, n (%)					0.600
0 year	3 (4.5)	3 (8.1)	0 (0.0)	0 (0.0)	
6–8 years	11 (16.4)	8 (21.6)	1 (8.3)	2 (11.1)	
9–13 years	22 (32.8)	12 (32.4)	3 (25.0)	7 (38.9)	
> 13 years	31 (46.3)	14 (37.9)	8 (66.7)	9 (50.0)	
Relationship status, n (%)					0.195
Single	25 (37.9)	11 (29.7)	5 (45.45)	9 (50.0)	
Married	26 (39.4)	16 (43.2)	6 (54.55)	4 (22.2)	
Divorced	1 (1.5)	1 (2.7)	0 (0.0)	0 (0.0)	
Widower	4 (6.0)	4 (10.8)	0 (0.0)	0 (0.0)	
Living with partner	10 (15.2)	5 (13.5)	0 (0.0)	5 (27.8)	
Socioeconomic status, n (%)					0.105
< 1000 euro/month	14 (20.9)	11 (29.7)	2 (16.7)	1 (5.6)	
> 1000 euro/month	53 (79.1)	26 (70.3)	10 (83.3)	17 (94.4)	
*Ticket exemption, n (%)					**0.015**
Not exempt	36 (52.2)	15 (38.5)	8 (66.7)	13 (72.2)	
Partially exempt	32 (46.4)	24 (61.5)	4 (33.3)	4 (22.2)	
Totally exempt	1 (1.4)	0 (0.0)	0 (0.0)	1 (5.6)	

*IQR* interquartile range

Is considered significant and bold typed when p < 0.025 after Bonferroni correction

^£^After Bonferroni correction there was a significant difference between EC and EoE (p = 0.003)

^&^After Bonferroni correction there was a significant difference between EC and IBS (p = 0.014)

^*^After Bonferroni correction there was a significant difference between EC and IBS (p = 0.007)

No difference was observed across the three groups with regards to socio-economic factors and educational status. Overall, most patients across the three groups were not exempt or partially exempt from healthcare payment.

### Clinical, histopathological, and laboratory data

Overall, the frequency of atopic comorbidities was not significantly different among the three groups (p = 0.64), as shown in Table 2. Analysing the specific clinical manifestations of allergy, relevant differences among the groups emerged with regard to respiratory allergy. While allergic rhinitis was more frequent in patients with IBS as compared to EC (p = 0.001), EC had a significantly higher frequency of patients with asthma as compared to IBS (p = 0.02). Despite not reaching statistical significance, food allergy, eczema, and drug allergy seemed to be more prevalent in patient with EC than IBS. Of note, autoimmune manifestations were far less frequent in EC as compared to IBS (p = 0.018).

**Table 2:** Comparison of symptom frequency in 40 eosinophilic colitis (EC) patients versus 12 eosinophilic esophagitis (EoE) patients and 21 irritable bowel syndrome (IBS) patients

Variable	Overall cohort (n = 73)	EC (n = 40)	EoE (n = 12)	IBS (n = 21)	p-value (trend)	p-value EC v/s EoE	p-value EC v/s IBS	p-value EoE v/s IBS
Atopic and autoimmune manifestations								
Atopy, n (%)					0.644			
Yes	56 (76.7)	31 (77.5)	8 (66.7)	17 (81.0)				
No	17 (23.3)	9 (22.5)	4 (33.3)	4 (19.0)				
Rhinitis, n (%)					**0.002**	0.094	**0.001**	0.42
Yes	39 (53.4)	14 (35.0)	8 (66.7)	17 (81.0)				
No	34 (46.6)	26 (65.0)	4 (33.3)	4 (19.0)				
Asthma, n (%)					**0.039**	0.47	**0.022**	0.53
Yes	16 (21.9)	13 (32.5)	2 (16.7)	1 (4.8)				
No	57 (78.1)	27 (67.5)	10 (83.3)	20 (95.2)				
Eczema, n (%)					0.245			
Yes	12 (16.4)	6 (15.0)	4 (33.3)	2 (9.5)				
No	61 (83.6)	34 (85.0)	8 (66.7)	19 (90.5)				
Food allergy, n (%)					0.435			
Yes	16 (21.9)	9 (22.5)	4 (33.3)	3 (14.3)				
No	57 (78.1)	31 (77.5)	8 (66.7)	18 (85.7)				
Drug allergy, n (%)					0.756			
Yes	12 (16.4)	8 (20.0)	1 (8.3)	3 (14.3)				
No	61 (83.6)	32 (80.0)	11 (91.7)	18 (85.7)				
Hymenoptera allergy, n (%)					1.000			
Yes	2 (2.7)	1 (2.5)	0 (0.0)	1 (4.8)				
No	71 (97.3)	39 (97.5)	12 (100)	20 (95.2)				
Anaphylaxis, n (%)					1.000			
Yes	2 (2.7)	1 (2.5)	0 (0.0)	1 (4.8)				
No	71 (97.3)	39 (97.5)	12 (100)	20 (95.2)				
Multiple autoimmune disease, n (%)					0.051			
Yes	8 (11.6)	3 (7.7)	0 (0.0)	5 (27.8)				
No	61 (88.4)	36 (92.3)	12 (100)	13 (72.2)				
Autoimmune disease > 1, n (%)					**0.037**	1.000	**0.018**	0.066
None	60 (87.0)	35 (89.7)	12 (100)	13 (72.2)				
Vitiligo	1 (1.4)	1 (2.6)	0 (0.0)	0 (0.0)				
Connectivitis (SLE, Sjogren etc.)	2 (2.9)	2 (5.1)	0 (0.0)	0 (0.0)				
Intestinal immune-mediated diseases (IBD, coeliac disease)	6 (8.7)	1 (2.6)	0 (0.0)	5 (27.8)				
Gastrointestinal manifestations								
Dyspeptic symptoms, n (%)					** < 0.001**	0.093	** < 0.001**	** < 0.001**
Present	32 (43.8)	21 (52.5)	10 (83.3)	1 (4.8)				
Absent	41 (56.1)	19 (47.5)	2 (16.7)	20 (95.2)				
Gastroesophageal reflux, n (%)					** < 0.001**	0.329	** < 0.001**	** < 0.001**
Present	27 (37.0)	19 (47.5)	8 (66.7)	0 (0.0)				
Absent	46 (63.0)	21 (52.5)	4 (33.3)	21 (100)				
Diarrhoea, n (%)					** < 0.001**	** < 0.001**	0.237	**0.004**
Present	45 (61.6)	31 (77.5)	1 (8.3)	13 (61.9)				
Absent	28 (38.4)	9 (22.5)	11 (91.7)	8 (38.1)				
Weight loss, n (%)					0.230			
Present	16 (21.9)	12 (30.0)	1 (8.3)	3 (14.3)				
Absent	57 (78.1)	28 (70.0)	11 (91.7)	18 (85.7)				
Pain/abdominal distention, n (%)					** < 0.001**	** < 0.001**	0.222	** < 0.001**
Present	46 (63.0)	28 (70.0)	0 (0.0)	18 (85.7)				
Absent	27 (37.0)	12 (30.0)	12 (100)	3 (14.3)				
Faecal occult blood, n (%)					**0.021**			
Present	9 (14.75)	9 (22.5)	–	0 (0.0)				
Absent	52 (85.25)	31 (77.5)	–	21 (100)				
Bowel movement number, median (IQR)	–	4 (2–6)	–	1 (1–2)	** < 0.001**			

*IBD* inflammatory bowel disease, *IQR* interquartile range, *SLE* systemic lupus erythematosus

P is considered significant and bold typed when p < 0.025 after Bonferroni correction

Analysing clinical symptoms and signs related to colon involvement, there were significant differences across the three groups regarding diarrhoea (p < 0.001), faecal occult blood positivity (p = 0.02), together with other gastrointestinal symptoms such as dyspepsia (p < 0.001; Table 2). The prevalence of abdominal pain/distention in EC was higher than that of patients with EoE (p < 0.001), but not statistically different from patients with IBS (p = 0.22). Constipation was overall a rare finding in patient with EC, found in only 4/36 patients. Gastroesophageal reflux symptoms were also a frequent finding in patients in EC.

Patients presenting with abdominal pain/distention had more frequently a diagnostic eosinophilic infiltrate in transverse and left colon segments, *i.e*., descending colon or sigma, (p = 0.036, p = 0.22, p = 0.02, respectively; Supplementary Tables 1 and 2). Endoscopically, in most cases lesions were present in the left colon; hyperaemia and erosions were the most common findings, while frank ulcers were very uncommon.

There were no associations between the involved colonic segment (both histologically and endoscopically) and the presence of atopy and sensitization to lipid transfer protein, LTP, (Supplementary Table 3). Similarly, there was no correlation between the extent and severity of the eosinophilic infiltrate and EC clinical activity or clinical remission (Supplementary Table 4).

With regard to biochemical parameters (Table 3), higher serum eosinophil levels were found in patients with EoE (p < 0.001) as compared to both the other groups, whereas EC patients did not display statistically different levels (p = 0.47) as compared to IBS patients. On the contrary, in EC patients, higher levels of C reactive protein (CRP) were observed (p < 0.001) as compared to both the other groups, together with faecal calprotectin (p = 0.004) as compared to IBS. No statistically significant difference was found regarding total eosinophilic cationic protein (ECP) and total IgE levels.

**Table 3:** Comparison of blood and stool tests in 40 eosinophilic colitis (EC) patients versus 12 eosinophilic esophagitis (EoE) patients and 21 irritable bowel syndrome (IBS) patients

Variable	EC (n = 40)	EoE (n = 12)	IBS (n = 21)	p-value (trend)	p-value EC v/s EoE	p-value EC v/s IBS	p-value EoE v/s IBS
Eosinophil absolute count (× 10^9^/L), median (IQR)	145 (60–231)	425 (270–660)	140 (115–190)	** < 0.001**	** < 0.001**	0.47	** < 0.001**
Percentage of eosinophils, median, (IQR)	2.30 (1.20–5.90)	6.55 (4.34–8.33)	2.10 (1.46–2.50)	**0.014**	**0.012**	0.11	** < 0.001**
CRP (mg/dl), median (IQR)	0.25 (0.15–2.00)	0.00 (0.00–0.25)	0.02 (0.00–0.19)	** < 0.001**	** < 0.001**	** < 0.001**	0.49
ECP (mcg/l), median (IQR)	30.65 (20–60.50)	34.95 (10.42–71.85)	19.45 (7.05–42.60)	0.440			
FCAL (mg/Kg), median (IQR)	41 (25–132)	–	7.40 (0.00–7.70)	**0.004**		**0.002**	
Total IgE (kU/L), median (IQR)	177 (66.6–501)	470 (103.2–534.0)	158 (56.50–567.50)	0.823			

*CRP* C reactive protein; *ECP* eosinophil cationic protein, *FCAL* fecal calprotectin, *IgE* immunoglobulin E, *IQR* interquartile range

P is considered significant and bold typed when p < 0.025 after Bonferroni correction

Analysing the prevalence of allergic sensitization of the patients in the three groups, despite the rate of positivity to food/respiratory allergens was generally more frequent in EoE and IBS, in EC 18% of patients displayed skin positive responses to any food and 26% positive specific IgE to at least one food (Tables 4 and 5). IgE positivity to at least one food was more frequently observed in patients with EC and EoE as compared to IBS (p < 0.001; Table 5). Moreover, analysing the sensitization profile to molecular allergens, detectable specific IgE to all the major classes of pan-allergens, such as PR-10, profilin and LTP, were observed in EC patients. Moreover, they displayed higher rates of sensitization to PR-10 and profilin as compared to IBS patients (p = 0.021 for both comparisons). Of note, LTP sensitization was found in patients with EC and EoE, but not in those with IBS.

**Table 4:** Comparison of positive skin prick-tests for food in patients with eosinophilic colitis (EC) versus eosinophilic esophagitis (EoE) and irritable bowel syndrome (IBS)

Variable	Overall cohort*	EC	EoE	IBS	p-value Trend	p-value EC v/s EoE	p-value EC v/s IBS	p-value EoE v/s IBS
Any food, n (%)					**0.010**	0.047	**0.012**	0.67
Positive	17 (36.2)	5 (18.5)	6 (54.6)	6 (66.7)				
Negative	30 (63.8)	22 (81.5)	5 (45.4)	3 (33.3)				
Wheat, n (%)					**0.001**	0.308	**0.002**	0.11
Positive	5 (10.4)	0 (0.0)	1 (8.3)	4 (44.4)				
Negative	43 (89.6)	27 (100)	11 (91.7)	5 (55.6)				
Peach, n (%)					0.479			
Positive	12 (25.0)	5 (18.5)	4 (33.3)	3 (33.3)				
Negative	36 (75.0)	22 (81.5)	8 (66.7)	6 (66.7)				
Apple, n (%)					0.667			
Positive	4 (6.7)	1 (3.7)	1 (8.3)	2 (9.5)				
Negative	56 (93.3)	26 (96.3)	11 (91.7)	19(90.5)				
Peanuts, n (%)					0.161			
Positive	6 (10.0)	2 (7.4)	3 (25.0)	1 (4.8)				
Negative	54 (90.0)	25 (92.6)	9 (75.0)	20 (95.2)				
Almond, n (%)					**0.006**	**0.024**	-	0.040
Positive	3 (5.0)	0 (0.0)	3 (25.0)	0 (0.0)				
Negative	57 (95.0)	27 (100)	9 (75.0)	21 (100)				
Hazelnut, n (%)					**0.023**	0.078	1.000	**0.040**
Positive	4 (6.7)	1 (3.7)	3 (25.0)	0 (0.0)				
Negative	56 (93.3)	26 (96.3)	9 (75.0)	21 (100)				
Walnut, n (%)					0.785			
Positive	3 (5.0)	1 (3.7)	1 (8.3)	1 (4.8)				
Negative	57 (95.0)	26 (96.3)	11 (91.7)	20(95.2)				
Soy, n (%)					0.138			
Positive	3 (5.0)	1 (3.7)	2 (16.7)	0 (0.0)				
Negative	57 (95.0)	26 (96.3)	10 (83.3)	21 (100)				
Shrimp, n (%)					**0.037**	0.089	-	0.125
Positive	2 (3.3)	0 (0.0)	2 (16.7)	0 (0.0)				
Negative	58 (96.7)	27 (100)	10 (83.3)	21 (100)				
Fish (cod, tuna, or salmon), n (%)	0 (0.0)	0 (0.0)	0 (0.0)	0 (0.0)	-			
Egg, yolk or albumen, n (%)					**0.037**	0.089	-	0.125
Positive	2 (3.3)	0 (0.0)	2 (16.7)	0 (0.0)				
Negative	58 (96.7)	27 (100)	10 (83.3)	21 (100)				
Milk, a-La, BLG, CAS, n (%)					1.000			
Positive	1 (1.7)	1 (3.7)	0 (0.0)	0 (0.0)				
Negative	59 (98.3)	26 (96.3)	12 (100)	21 (100)				

*a-La* alpha-lactalbumin; *BLG* beta-lactoglobulin; *CAS* casein; *CRP* C reactive protein; *FCAL* faecal calprotectin; *SPT* skin prick test

^*^Percentage calculated only in the cohort from Pavia and Padua

**Table 5:** Comparison of serum allergen specific-immunoglobulin E (IgE) in patients with eosinophilic colitis (EC) versus eosinophilic esophagitis (EoE) and irritable bowel syndrome (IBS)

Variable	EC*	EoE	IBS	p-value (trend)	p-value EC v/s EoE	p-value EC v/s IBS	p-value EoE v/s IBS
Positivity of at least 1 food, n	7/27	6/8	6/21	** < 0.001**	**0.032**	**0.014**	** < 0.001**
Wheat, gluten or gliadin, n	1/23	2/6	0/3	0.100			
Corn, n	0/22	1/4	6/15	**0.003**	0.154	**0.002**	1.000
Rice, n	0/22	1/4	4/10	**0.005**	0.154	**0.006**	1.000
Milk, a-LA, b-LG or CAS, n	1/22	1/6	3/11	0.110			
Egg, yolk or albumen, n	0/22	1/6	2/13	0.122			
Nut (walnut or hazelnut), n	4/22	4/8	0/13	**0.018**	0.158	0.274	**0.012**
Crustacean (shrimp or crab) n	0/21	1/6	1/13	0.219			
Fish (cod, tuna or salmon), n	0/22	1/7	2/10	0.074			
Soy, n	0/22	3/7	2/10	**0.008**	**0.010**	0.091	0.593
Molecular allergens							
PR-10 (*Bet v 1*), n	5/20	4/7	2/21	**0.001**	0.175	**0.021**	**0.002**
Profillin (*Phl p12 or Bet v 2),* n	5/20	2/7	0/21	**0.021**	1.000	**0.021**	0.056
Peach LTP, (*Pru p 3*) n	4/26	5/9	0/5	**0.024**	0.030	1.000	0.086
Peanut LTP (*Ara h 9*), n	2/15	3/4	2/5	**0.032**	0.037	0.249	0.524
Wheat LTP (*Tri a 14*), n	1/14	1/3	0/10	0.214			
Hazelnut LTP (*Cor a 8*), n	0/13	3/4	0/7	**0.002**	**0.006**	-	**0.024**
Walnut LTP (*Jug r 3*), n	3/14	3/4	0/3	0.068			
*Pru p 3,* median (IQR)	2.02 (0.48–7.40)	4.34 (0.0–10.0)	0.0 (0.0–0.0)	0.097			
*Ara h 9*, median (IQR)	4.18 (0.2–8.16)	1.0 (0.0–1.0)	0.0 (0.0–0.0)	0.129			
*Jug r 3*, median (IQR)	0,74 (0.14–1.34)	0.22 (0.0–9.8)	0.0 (0.0–0.0)	0.270			

*aLA* alpha-lactalbumin; *bLG* beta-lactoglobulin, *CAS* casein, *IQR* interquartile range; *LTP* lipid transfer protein; *PR-10* pathogenesis related-protein 10. IgE serum levels > 0.1 kUa/L by FEIA were considered positive. *Proportions were calculated only for the cohort of Pavia and Padua

Finally, comparing positive skin prick tests and specific IgE in patients with EC we found no sensitizations to allergens of animal origin, such as milk, egg of fish, except in one patient (milk).

Therapeutical agents prescribed in patients with EC were as follows: rifaximin (n = 4, 10%), mesalazine (n = 15, 37.5%), probiotics (n = 10, 25%), budesonide (n = 16, 60%), systemic corticosteroids (n = 5, 12.5%), antidiarrheal agents (n = 8, 20%), combination therapy (budesonide ± other therapies; n = 18, 45%). More than one therapy was given in most cases. None of the patients was taking an immunosuppressant.

### Diagnostic delay and misdiagnosis

In EC the median overall diagnostic delay was 10 months (IQR 4–15), the median patient-dependent diagnostic delay was 3 months (IQR 1–6), and the median physician-dependent diagnostic delay was 6 months (IQR 3–12). Diagnostic delay was mostly physician-related and did not vary according to gender, biochemical parameters, and most clinical symptoms, except for weight loss (Table 6). Additionally, factors significantly contributing to a greater diagnostic delay were atopy for the patient-dependent delay, weight loss for the physician-dependent and overall delays, and a previous misdiagnosis for physician-dependent delay. The most frequent misdiagnoses were IBS (n = 12), followed by IBD (n = 10), eosinophilic granulomatosis with polyangiitis (n = 2), and unspecific colitis (n = 1).

**Table 6:** Association between diagnostic delay and demographics, clinical and laboratory tests in patients with eosinophilic colitis*

Variable	Patient-dependent delay (months)	Physician-dependent delay (months)	Overall delay (months)
Gender, median (IQR)			
Female	3 (2–6)	8 (3–12)	10 (6–14)
Male	1.5 (7–1)	4.5 (2.5–18)	10 (3–29.5)
Previous misdiagnosis, median (IQR)			
Yes	3 (1–8)	**8 (3–12)**	12 (6–24)
No	1 (2–3)	3.5 (2–8)	9 (4–13)
Atopy, median (IQR),			
Present	**3 (2–8)**	6 (3–12)	10 (6–24)
Absent	1 (1–2)	4.5 (1.5–10)	6.5 (3–11.5)
Autoimmune disease, median (IQR)			
More than 1 disease	3 (1–6)	6 (3–24)	10 (4–15)
At least one or no other disease	3 (1–3)	6 (3–12)	9 (4–27)
Serum eosinophil Count (× 10^9^/L), median (IQR)			
> 150 × 10^9^/L	5 (2–8)	6 (3–12)	13 (6–24)
< 150 × 10^9^/L	2 (1–3)	6 (2.5–10)	10 (4–12.5)
FCAL (mg/Kg), median (IQR)			
> 50 mg/Kg	3 (2–6.5)	9 (4–12)	11.5 (7.5–19.5)
< 50 mg/Kg	2 (1–6)	5 (3–8)	9 (4–13)
Total IgE (kU/L), median (IQR)			
> 100 kU/L	5.5 (2–10)	9 (6–12)	14.5 (10–25)
< 100 kU/L	3.5 (2–8)	5.5 (4–9)	9 (6.5–10.5)
Dyspepsia, median (IQR)			
Present	2 (1–5)	6 (3–12)	10 (4–14)
Absent	3 (1–8)	6 (3–12)	10 (6–18)
Gastroesophageal reflux, median (IQR)			
Present	2 (1–4)	6 (3–10)	10 (4–12)
Absent	3 (1–10)	7 (3–12)	13.5 (6–25)
Diarrhoea, median (IQR)			
Present	3 (1–6)	6 (3–12)	10 (6–15)
Absent	2.5 (1–9)	6.5 (3–12)	9 (3.5–18.5)
Weight loss, median (IQR)			
Present	4 (2–7)	**12.5 (7–24)**	**20 (10–29.5)**
Absent	2 (1–6)	5 (3–10)	9 (4–13)
Abdominal pain/distention, median (IQR)			
Present	2.5 (1–4)	8 (3–12)	10 (6–15)
Absent	6 (1–10)	4 (3–6)	6 (4–14)
Constipation, median (IQR)			
Present	4.5 (3–5.5)	9 (6.5–23)	12.5 (9.5–28.5)
Absent	2 (1–6)	6 (3–12)	10 (4–14)
Faecal occult blood, median (IQR)			
Positive	1 (1–2)	3 (2–6)	7 (2–10)
Negative	3 (2–6)	8 (3–12)	10 (6–15)

^*^Data available only for the cohort of Pavia and Torino

*FCAL* faecal calprotectin; *IgE* immunoglobulin E; *IQR* interquartile range

Significant test p-value < 0.05 are shown in bold type

### Factors associated with EC relapse

Regarding factors associated with the disease status (active disease or disease remission) in the Pavia cohort at the time of the last evaluation, the positivity of skin prick test and specific IgE for food and/or inhalants did not discriminate active patients from patients in remission (Supplementary Table 5). The presence of positivity of skin prick tests or specific IgE for food (p = 0.021, p = 0.05, respectively), but not skin positivity to inhalants (p = 0.065), was associated with a relapsing disease course.

## Discussion

In this retrospective multicentre study involving four Italian tertiary referral centres for the management of primary EGID, we showed that adult EC patients, who are mostly female, are burdened by a high prevalence of atopic comorbidity, particularly allergic rhinitis, and asthma, and by a substantial diagnostic delay, particularly in those who had been previously misdiagnosed. While peripheral blood eosinophil count was increased in EoE, but not EC, in EC faecal calprotectin discriminated them from IBS. Finally, no clear correlation between the site and entity of the eosinophilic gut infiltration and clinical manifestations or the risk of relapse was noticed in EC. To the best of our knowledge, our cohort of adult patients with primary EC is the largest described so far by adopting the more stringent histological cut-off values [11], and well-studied from an allergy point of view. No similar studies looking at a wide allergy characterisation are in fact available, nor have data about the diagnostic delay in EC been published so far. Moreover, our series of EC patients was compared with age-matched control groups. Consistently with other series, as expected, we observed a higher prevalence of female sex in EC patients, as opposed to EoE, and a predominant mucosal involvement [6, 19]. Yet, several other novel data emerged in our study.

Considering putative risk factors, smoking, defined as a current or previous smoking habit, was much more prevalent in EC patients than in patients with IBS. Moreover, family history of EC did not have a relevant role in our series. These findings are of interest and may be disease-specific features, since EoE, on the other hand, is generally characterized by familial aggregation of cases and smoking does not seem to have a role [20]. While a certain association between smoking and IBS has been demonstrated [21], no data about smoking in EC are available. Indeed, due to the limited sample size, more studies are needed to confirm an association between smoking and EC.

Most patients with EC in our series were exempt from healthcare payment. Lower income has been associated with poorer diet quality, *i.e.,* a diet with poor of vegetable and fruits and rich in saturated fats and sugar, and thus containing fewer micronutrients and less fibre [22, 23]. It is possible that diet-related factors including alterations of the microbiota may have a role in determining or worsening EC. In this instance, it has been shown that probiotic supplementation contributes to eosinophil trafficking and inflammation in the colon [24, 25]. In our series, we could not retrieve precise data about the dietary habits and the socioeconomic status, and hence more studies are needed in this regard.

In contrast to other series, however limited by the small sample size, the frequency of atopy in EC was more relevant, reaching 77%, with a frequency of asthma and food allergy of 32% and 25%, respectively. In a Spanish retrospective study by Diaz Del Arco enrolling 22 cases of EC among 106 cases of colonic eosinophilia, the prevalence of atopy was overall low, with a rate of asthma of 6.6%, and that of food allergy of 2.8% [19]. The high prevalence of atopic comorbidity may partly be related to the thorough allergy screening that was carried out in our patients, and to the poor allergy characterisation in the available, retrospective series. Yet, the fact that a specific association between EC and asthma was found*,* while IBS was more frequently associated with rhinitis, seems to underlie different pathogenetic pathways that warrant further attention, including a shared (epi)genetic background between EC and Th-2 respiratory inflammation. On the other hand, autoimmunity was more frequently found to be associated with IBS, as compared to eosinophilic disorders. This finding may either underlie a common pathogenetic element (*i.e.*, an immune perturbation involving the gut) or may simply reflects the extensive autoimmune screening patients with IBS usually undergo (*e.g.,* autoimmune thyroid disease, coeliac disease), since IBS is actually a diagnosis of exclusion.

The main clinical presentation of EC, consistently with other series, is diarrhoea with pain and abdominal distension, reflecting the mucosal form of the colon involvement, which is the most frequent in this disease [2, 3]. Yet, the diagnosis of EC is particularly challenging, given the lack of major classifying clinical manifestations, evocative endoscopic features (i.e., unspecific or mild findings in most cases), and EC-specific diagnostic biomarkers. Indeed, the most frequently suspected disease before colonoscopy was IBD. Moreover eosinophil-related markers, such as eosinophil counts and ECP, did not differentiate EC patients form IBS in our series. Finally, the presence of a generic allergic comorbidity per se was not able to discriminate between EC and IBS, and these are also frequently found in IBD [26]. The only discriminating comorbidities between EC and IBS were asthma (more common in the former) and rhinitis (more common in the latter). Thus, the diagnosis of EC, also on the basis of our data, is still a diagnosis of exclusion and relies on histology, since endoscopic features are not pathognomonic. Indeed, possibility of this disorder should be considered in patients presenting with diarrhoea or other abdominal symptoms, weight loss, a positive faecal calprotectin, particularly when endoscopic, and other histopathological features of IBD are absent.

Analysing the diagnostic delay in EC, we found a significant overall diagnostic delay of 10 months (IQR 4–15), which was mostly related to the physician-dependent diagnostic component than the patient-related one. The most frequent misdiagnoses were IBS and IBD. This result is similar to other organic gastrointestinal disorders, in which a misdiagnosis with other disorders mimicking the “true" organic disease is very common, including autoimmune gastritis (often misdiagnosed with functional dyspepsia), EoE (often misdiagnosed with gastroesophageal reflux disease), IBD (often misdiagnosed with IBS), and coeliac disease (often misdiagnosed with IBS) [27–30]. For all the abovementioned gastrointestinal conditions, the diagnostic delay has been found to be substantial in all cases, but possibly reducing over the last decades due to a better awareness of those conditions and the wider use of endoscopy as a first-line examination. It can be speculated that in EC, in the setting of weight loss, physicians may initially prescribe diagnostic tests other than colonoscopy, such as imaging test, including abdominal ultrasound and computer tomography, or colonoscopy may be performed without biopsies thus further delaying the diagnosis. On the other hand, it is possible that patients initially attribute their clinical manifestations to food allergy and start self-imposed dietary modifications before seeking medical evaluation. However, although substantial, the diagnostic delay in EC was found to be much shorter than that of EoE, in which the median overall diagnostic delay was 36 months [29]. There could be some clinical factors or features contributing to a generally lower delay in EC compared to other gastrointestinal disorders, such as the availability of a stool inflammatory marker (i.e., faecal calprotectin), though unspecific; the constantly present symptoms, in many cases alarming; and the absence of compensatory eating behaviours, such as avoidance of certain foods causing dysphagia in EoE, slow eating, and food texture modification.

Analysing the frequency of sensitization to allergens, we observed several novel findings. First, an overall lower rate of skin prick test positivity, as opposed to IBS, was found though 18% of patients displayed skin positive responses to any food and 26% positive specific IgE to at least one food. Of note, IBS patients displayed more frequent sensitization to wheat, possibly reflecting cross-reactivity with grasses, which is a common trigger of rhinitis.

Interestingly, in EC patients we did not detect evidence of sensitization to animal proteins, such as milk, meat, egg, except for one patient sensitized to milk. This is in stark contrast to EoE, where IgE responses/allergy to milk and egg is frequently found, particularly in paediatric patients [31]. Moreover, we showed that EC patients displayed sensitization to several classes of pan-allergens, such as PR-10, profilin, and LTP. The role of thermo- and acid-labile protein, such as PR-10 and profilin, as food allergens, is unlikely to be relevant in the pathogenesis of colon inflammation since they are totally degraded in upper segments of the gastrointestinal tract, while their positivity may simply reflect the co-occurrence of respiratory allergy. On the contrary, LTP, is a “true” food allergen, due to its stability to temperature, acidic environment, and proteases (such as pepsin), and main gastrointestinal route of sensitization, and may directly play a role, at least in a subset of patients in EC in the Mediterranean area, where it is geographically diffuse, as we have shown for other EGID [32, 33]. Finally, we showed in a part of our cohort where complete data were available, that patients sensitized to food allergens had a higher number of disease relapse. It is possible that the chronic immune stimulation due to food antigens and contribute to the ongoing gut inflammatory process. If replicated in our series, this finding may have significant implications in the management of EC patients as those with allergic sensitization may therefore benefit from a more careful observation in the follow-up, after an acute episode has resolved.

Our study has some limitations that should be mentioned. The retrospective nature has all the intrinsic limitations of such a design, especially regarding the lack of a standardised work-up across the four enrolling centres. The small sample size, particularly of the EoE did not allow to elicit significant differences with respect to EC, unless the effect size was exceedingly high (given a type I error of 5% and a power of 80%). However, our study was descriptive in nature and the statistical comparisons could only suggest the relative importance of the different features analysed in the three cohorts. Further, there was a sub-optimal availability of some allergology parameters, and mostly limited to the Pavia centre, which possibly reflects the seldom diffuse practice among physicians caring for EC patients to recommend a thorough allergology evaluation. We tried to overcome this issue by excluding missing data from percentage calculation and by only comparing patients who actually completed the allergology evaluation. Indeed, some variables that were collected through a phone call may be open to recall biases; however, we tried to overcome this limitation by asking only for verifiable information (e.g., socioeconomic status). For this reason, data about smoking and alcohol consumption could not be more precise (e.g., actual number of smoked cigarettes, precise amount of alcohol consumption, etc.…).

Indeed, we are aware that larger confirmatory studies are needed in order to strengthen our findings. Nonetheless, some strengths should be mentioned as well, including the largest sample of the cohort described so far, the details provided, and the strict pathological diagnostic criteria adopted for all conditions.

In conclusion, in the lack of any major classifying clinical manifestation and of EC-specific diagnostic biomarkers, the copresence of atopy or ongoing/refractory clinical symptoms especially in female patients, along with the elevation of faecal calprotectin should raise the suspicious of EC. Future prospective studies are needed to better ascertain the relationship between atopy and EC. Finally, future clinical trials should consider the complete lack of association between histopathological severity and clinical symptoms to tailor proper endpoints. Under this point of view, as we had already hypothesised in EoE [34], the eosinophil should not be the only therapeutic target.

### Supplementary Information

Below is the link to the electronic supplementary material.

Supplementary file1 (DOCX 13 KB)

Supplementary file2 (DOCX 21 KB)

Supplementary file3 (DOCX 21 KB)

Supplementary file4 (DOCX 18 KB)

Supplementary file5 (DOCX 17 KB)

Supplementary file6 (DOCX 18 KB)

## Data Availability

All data pertaining the study have been presented in the manuscript. Additional data can be asked to the corresponding author.

## Abbreviations

EC: Eosinophilic colitis
EGID: Eosinophilic gastrointestinal disorders
EoE: Eosinophilic esophagitis
IBS: Irritative bowel syndrome
