# Supplementary material for: Clinical and atopic features of patients with primary eosinophilic colitis: an Italian multicentre study
Source: Intern Emerg Med. 2024 Mar 10;19(4):993–1005. doi: 10.1007/s11739-024-03568-w (PMC11186925; doi:10.1007/s11739-024-03568-w)
Supplement: Supplementary file 1 — Supplementary file1 (DOCX 13 KB) [file 11739_2024_3568_MOESM1_ESM.docx]

**Supplementary table statistics.** Minimal detectable difference (effect size) between the EC cohort (40 patients) and EoE (12 patients) or IBS (21 patients) in a series of potential scenarios, when the type I error is 5% and the power 80%.

| **Proportion with feature**  **in EC (N=40)** | **vs CE (N=12)**  **minimal detectable difference**  **(effect size)** | **vs IBS (N=21)**  **minimal detectable difference**  **(effect size)** |
| --- | --- | --- |
| 0.20 | 0.44 | 0.36 |
| 0.30 | 0.44 | 0.37 |
| 0.40 | 0.42 | 0.36 |
| 0.50 | 0.39 | 0.34 |
| 0.60 | 0.34 | 0.31 |
| 0.70 | 0.29 | 0.26 |
