# Supplementary material for: Clinical and atopic features of patients with primary eosinophilic colitis: an Italian multicentre study
Source: Intern Emerg Med. 2024 Mar 10;19(4):993–1005. doi: 10.1007/s11739-024-03568-w (PMC11186925; doi:10.1007/s11739-024-03568-w)
Supplement: Supplementary file 2 — Supplementary file2 (DOCX 21 KB) [file 11739_2024_3568_MOESM2_ESM.docx]

**Supplementary Table 1.** Relationship between clinical manifestations in patients with eosinophilic colitis and site of diagnostic eosinophilic infiltrate on histology and endoscopic appearance.

|  | **Atopy** | **Dyspepsia** | **Gastroesophageal**  **reflux** | **Diarrhea** | **Weight**  **loss** | **Abdominal pain/**  **distention** | **Constipation** | **Faecal stool**  **blood** |
| --- | --- | --- | --- | --- | --- | --- | --- | --- |
| **Diagnostic intestinal segment, n, (%)** | | | | | | | | |
| Caecum | 10 (71.4) | 4 (28.5) | 3 (21.4) | 11 (78.6) | 3 (21.4) | 11 (78.6) | 2 (14.3) | 1 (7.1) |
| Right colon | 10 (83.3) | 5 (41.7) | 5 (41.7) | 9 (75) | 3 (25) | 8 (66.7) | 2 (16.7) | 0 (0) |
| Transverse colon | 7 (70) | 1 (10) | 3 (30) | 8 (80) | 4 (40) | 5 (50), **p 0.036** | 2 (20) | 0 (0) |
| Left colon | 5 (83.3) | 0 (0) | 2 (33.3) | 5 (83.3) | 3 (50) | 2 (33.3), **p 0.022** | 1 (16.7) | 0 (0) |
| Sigma | 4 (80) | 0 (0) | 0 (0) | 4 (80) | 1 (20) | 1 (20), **p =0.002** | 0 (0) | 0 (0) |
| Rectum | 4 (80) | 0 (0) | 0 (0) | 5 (100) | 1 (20) | 2 (40) 0.117 | 0 (0) | 1 (20) |
| **Endoscopic appearance** | | | | | | | | |
| Right colon lesions, n, (%), | 4 (57.1) | 6 (85.7) | 6 (85.7) | 4 (57.1) | 1 (14.3) 0.642 | 5 (71.4) | 1 (14.3) | 1 (14.3) |
| Hyperemia | 1 (33.3) | 2 (66.7) | 2 (66.7) | 1 (33.3) | 0 (0) 0.550 | 1 (33.3) | 0 (0) | 0 (0) |
| Erosion | 3 (50) | 5 (83.3) | 5 (83.3) | 3 (50) | 1 (16.7) 1.000 | 4 (66.7) | 1 (16.7) | 1 (16.7) |
| Ulcer | - | - | - | - | - | - | - | - |
| Edema | - | - | - | - | - | - | - | - |
| No vascular patterns | - | - | - | - | - | - | - | - |
| Left colon lesions, n, (%) | 7 (63.6) | 7 (63.6) | 7 (63.6) | 7 (63.6) | 3 (27.3) | 8 (72.7) | 1 (9) | 2 (18.2) |
| Hyperemia | 4 (66.7) | 2 (33.3) | 2 (33.3) | 4 (66.7) | 2 (33.3) | 4 (66.7) | 0 (0) | 1 (16.7) |
| Erosion | 3 (60) | 4 (80) | 4 (80) | 3 (60) | 2 (40) | 4 (80) | 0 (0) | 2 (40) |
| Ulcer | 1 (100) | 0 (0) | 0 (0) | 1 (100) | 1 (100) | 1 (100) | 0 (0) | 0 (0) |
| Edema | - | - | - | - | - | - | - | - |
| No vascular patterns | 1 (100) | 1 (100) | 1 (100) | 1 (100) | 0 (0) | 1 (100) 1 | 1 (100) | 0 (0) |

Only significant p values are shown in bold type.

Diagnostic cutoff for eosinophilic infiltrate were: > 100/HPF for caecum and right colon, >84/HPF for transverse and left colon, >64/HPF for sigma and rectum

Abbreviations: IQR, interquartile range.
