# Supplementary material for: Clinical and atopic features of patients with primary eosinophilic colitis: an Italian multicentre study
Source: Intern Emerg Med. 2024 Mar 10;19(4):993–1005. doi: 10.1007/s11739-024-03568-w (PMC11186925; doi:10.1007/s11739-024-03568-w)
Supplement: Supplementary file 3 — Supplementary file3 (DOCX 21 KB) [file 11739_2024_3568_MOESM3_ESM.docx]

**Supplementary Table 2.** Median eosinophil infiltrate according to different clinical manifestations and site of the colon and rectum

| **Variable**  **(median, IQR)** | **Caecum** | **Right colon** | **Transverse** | **Left colon** | **Sigma** | **Rectum** |
| --- | --- | --- | --- | --- | --- | --- |
| **Atopy** |  |  |  |  |  |  |
| Present | 101 (100-101) | 101 (70-107) | 81.5 (46-86) | 56.5 (30-85) | 43 (25-65) | 23.5 (15.5-65) |
| Absent | 110.5 (101.5-119.5) | 95.5 (78-118) | 93 (81.5-101) | 47.5 (12-84.5) | 24 (21.5-63) | 36 (22-56) |
| **Dyspepsia** |  |  |  |  |  |  |
| Present | 101 (100-116) | 104 (101-114) | 59 (53-78) | 37 (24-53) | 43 (28-55) | **12 (7-19)** |
| Absent | 101 (101-106) | 101 (70-101) | 85 (78-101) | 80 (30-89) | 27.5 (20-85.5) | **47 (25-65)** |
| **GERD** |  |  |  |  |  |  |
| Present | 100 (77-116) | 101 (60-140) | 81.5 (59-85) | 56.5 (37-85) | 43 (25-55) | **19 (12-20)** |
| Absent | 101 (101-106) | 101 (80-101) | 85 (44.5-98) | 53.5 (14.5-87) | 29 (21.5-85.5) | **57 (25-65)** |
| **Diarrhea** |  |  |  |  |  |  |
| Present | 101 (101-116) | 101 (66-114) | 85 (59-95) | 60 (30-85) | 30 (23-65) | 27 (19.5-65) |
| Absent | 101 (89-101) | 101 (93-101) | 64 (42.5-93) | 33.5 (13-71) | 41.5 (22-78) | 22 (11-36) |
| **Weight loss** |  |  |  |  |  |  |
| Present | 100 (77-101) | 80 (43-101) | 85 (78-101) | 69 (37-101) | 25 (20-55) | 19.5 (13-60) |
| Absent | 101 (101-117) | 101 (93-107) | 81.5 (49.5-90.5) | 53.5 (14.5-82.5) | 36.5 (24-67.5) | 27 (20.5-65) |
| **Abdominal pain/distention** |  |  |  |  |  |  |
| Present | 101 (101-116) | 101 (66-107) | **78 (46-85)** | **37 (15-60)** | **25 (20-43)** | **21 (15.5-27)** |
| Absent | 101 (80.5-101) | 101 (90-101) | **95 (86-101)** | **89 (85-112)** | **85.5 (67.5-101)** | **65 (56-83)** |
| **Constipation** |  |  |  |  |  |  |
| Present | 88.5 (68.5-115.5) | 80.5 (50.5-120.5) | 81.5 (65.5-85) | 56.5 (45-72.5) | 40 (22.5-55.5) | 19.5 (13-21) |
| Absent | 85 (46-101) | 101 (90-107) | 85 (46-101) | 53.5 (15-89) | 30 (23-70) | 38 (22-65) |
| **Faecal stool blood** |  |  |  |  |  |  |
| Positive | 110 (110-110) | 43 (43-43) | 24 (24-24) | 30 (30-30) | 10 (10-10) | 100 (100-100) |
| Negative | 110 (110-109) | 101 (90-107) | 85 (59-95) | 60 (24-85) | 36.5 (24-67.5) | 25 (19-65) |

Diagnostic cutoff for eosinophilic infiltrate were: > 100/HPF for caecum and right colon, >84/HPF for transverse and left colon, >64/HPF for sigma and rectum

Abbreviations: GERD, gastroesophageal reflux; IQR, interquartile range.

Significant tests, p-value < 0.05, are written in bold type.
