# Supplementary material for: Clinical and atopic features of patients with primary eosinophilic colitis: an Italian multicentre study
Source: Intern Emerg Med. 2024 Mar 10;19(4):993–1005. doi: 10.1007/s11739-024-03568-w (PMC11186925; doi:10.1007/s11739-024-03568-w)
Supplement: Supplementary file 4 — Supplementary file4 (DOCX 18 KB) [file 11739_2024_3568_MOESM4_ESM.docx]

**Supplementary Table 3.** Clinical, histological and endoscopical features of patients with atopic or positive for peach LTP (*Pru p 3)*.

| **Variable** | **Atopy** | **Peach LTP positivity** |
| --- | --- | --- |
| **Diagnostic intestinal segment, (%), p-value** | | |
| Caecum | 10 (71.4), 0.524 | 13 (7.7), 0.121 |
| Right Colon | 10 (83.3), 0.603 | 11 (18.2), 1.000 |
| Transverse colon | 7 (70), 0.588 | 10 (20), 1.000 |
| Left colon | 5 (83.3), 1.000 | 6 (0), 0.515 |
| Sigma | 4 (80), 1.000 | 5 (20), 1.000 |
| Rectum | - | 5 (20), 1.000 |
| **Histologic eosinophilic infiltrate, median, (IQR), p-value** | | |
| Caecum | 101 (100-101) 0.091 | 77 (60-101), 0.078 |
| Right Colon | 101 (70-107) 0.878 | 101 (70-107), 1.000 |
| Transverse colon | 81.5 (46-86) 0.231 | 85 (42-95), 0.785 |
| Left colon | 56.5 (30-85) 0.557 | 53 (12-60), 0.379 |
| Sigma | 43 (25-65) 0.605 | 55 (28-70), 0.585 |
| rectum | 23.5 (15.5-65) 0.748 | 19 (3-65), 0.386 |
| **Endoscopic appearance, n, (%), p-value** | | |
| RC lesions | 4 (57.1) 0.335 | 3 (0) 1.000 |
| Hyperemia | 1 (33.3) 0.156 | 1 (0) 1.000 |
| Erosion | 3 (50) 0.161 | 3 (0) 1.000 |
| Ulcus | - | - |
| Edema | - | - |
| No vascular pattern | - | - |
| LC lesions | 7 (63.6) 0.405 | 6 (0) 0.515 |
| Hyperemia | 4 (66.7) 0.634 | 4 (0) 0.541 |
| Erosion | 3 (60) 0.583 | 2 (0) 1.000 |
| Ulcer | 1 (100) 1.000 | 1 (0) 1.000 |
| Edema | - | - |
| No vascular patterns | 1 (100) 1.000 | 1 (0) 1.000 |

Abbreviations: IQR, interquartile range, LTP, lipid transfer protein.
