# Supplementary material for: Clinical and atopic features of patients with primary eosinophilic colitis: an Italian multicentre study
Source: Intern Emerg Med. 2024 Mar 10;19(4):993–1005. doi: 10.1007/s11739-024-03568-w (PMC11186925; doi:10.1007/s11739-024-03568-w)
Supplement: Supplementary file 5 — Supplementary file5 (DOCX 17 KB) [file 11739_2024_3568_MOESM5_ESM.docx]

**Supplementary Table 4.** Disease activity compared with diagnostic site of involvement and eosinophilic infiltrate in patients with eosinophilic colitis*.

| **Variable** | **Patient number (n=16)** | **Acute phase** | **Clinical remission** | **P-value** |
| --- | --- | --- | --- | --- |
| **Diagnostic site of involvement, n (%)** | | | | |
| Caecum | 13 | 4 (31.8) | 9 (69.2) | 1.000 |
| Right colon | 9 | 3 (33.3) | 6 (66.7) | 1.000 |
| Transverse colon | 9 | 3 (33.3) | 6 (66.7) | 1.000 |
| Left colon | 5 | 1 (20) | 4 (80) | 1.000 |
| Sigma | 5 | 1 (20) | 4 (80) | 1.000 |
| Rectum | 5 | 3 (60) | 2 (40) | 0.321 |
| **Eosinophilic infiltrate, median eosinophilic count/HPF, (IQR)** | | | | |
| Caecum |  | 100.5 (88.5-105.5) | 101 (101-102) | 0.290 |
| Right colon |  | 72 (42-101) | 101 (70-107) | 0.444 |
| Transverse colon |  | 81.5 (51-85) | 85 (46-95) | 0.333 |
| Left colon |  | 45 (33-82) | 60 (14-101) | 0.844 |
| Sigma |  | 40 (17.5-78) | 30 (23-70) | 0.519 |
| Rectum |  | 59.5 (13-100.5) | 25 (20-47) | 0.580 |

Abbreviations: HPF, high power field, IQR, interquartile range.

*Data were available from the centre of Pavia.
