# Supplementary material for: Clinical and atopic features of patients with primary eosinophilic colitis: an Italian multicentre study
Source: Intern Emerg Med. 2024 Mar 10;19(4):993–1005. doi: 10.1007/s11739-024-03568-w (PMC11186925; doi:10.1007/s11739-024-03568-w)
Supplement: Supplementary file 6 — Supplementary file6 (DOCX 18 KB) [file 11739_2024_3568_MOESM6_ESM.docx]

**Supplementary Table 5.** Allergological variables, disease status and disease flares in the Pavia cohort.

| **Variable** | **Active disease** | **In remission** | **P-value** |
| --- | --- | --- | --- |
| SPT for at least one inhalant, *n (%)* |  |  | 0.088 |
| Positive | 4 (44.4) | 5 (55.6) |  |
| Negative | 0 (0) | 6 (85.7) |  |
| SPT for at least one food, n (%) |  |  | 0.312 |
| Positive | 2 (66.7) | 1 (33.3) |  |
| Negative | 2 (15.4) | 10 (76.9) |  |
| IgE test for at least one food, n (%) |  |  | 0.234 |
| Positive | 3 (50) | 3 (50) |  |
| Negative | 1 (10) | 8 (80) |  |
|  | **Number of flares (median, IQR)** | |  |
| SPT for at least one inhalant |  |  | 0.065 |
| Positive | 3 (2-5) |  |  |
| Negative | 1 (1-1.5) |  |  |
| SPT for at least one food |  |  | **0.021** |
| Positive | 7 (5-8) |  |  |
| Negative | 1 (1-3) |  |  |
| IgE for at least one food |  |  | **0.005** |
| Positive | 4.5 (3-7) |  |  |
| Negative | 1 (1-2) |  |  |

Abbreviations: IgE: immunoglobulin E; SPT: skin prick test. Positivity was defined when at least one inhalant or food was positive and more precisely when the skin prick test was > 3 mm; allergen- specific serum IgE was considered positive when >0.1 kUa/l by FEIA.
